# Social Isolation and Aging Out of Place Among Immigrants and Refugee Seniors in Canada

**DOI:** 10.1093/geroni/igab046.1664

**Published:** 2021-12-17

**Authors:** Shanthi Johnson, Juanita-Dawne Bacsu, Tom McIntosh, Bonnie Jeffery, Nuelle Novik

**Affiliations:** 1 University of Alberta, Edmonton, Alberta, Canada; 2 University of Saskatchewan, Saskatoon, Saskatchewan, Canada; 3 University of Regina, Regina, Saskatchewan, Canada

## Abstract

Immigrant and refugee seniors experience cultural barriers, discrimination, and limited networks which increase the risk of social isolation and thus hinder their active participation in the society. This paper explores social isolation among immigrant and refugee seniors in Canada based on an environmental scan of federal/provincial/territorial and community-based programs and a systematic scoping review. Findings revealed important gaps and regional disparities in opportuntiies to reduce social isolation and great active participation. Research was limited, often qualitative in nature, typically based on larger urban centres, with measurement issues related to the need for consideration beyond one's living arrangements. The results highlight the need for greater understanding Canada’s immigration and refugee system and policies, and collaboration across levels of government. Reducing issues of social isolation and enabling better active aging for vulnerable seniors require a more nuanced and multidimensional conceptualization with prioritization on addressing the unique factors of culture and geographical context.

